# Supplementary material for: Human ALPI deficiency causes inflammatory bowel disease and highlights a key mechanism of gut homeostasis
Source: EMBO Mol Med. 2018 Mar 22;10(4):e8483. doi: 10.15252/emmm.201708483 (PMC5887907; doi:10.15252/emmm.201708483)
Supplement: Supplementary file 1 — Appendix [file EMMM-10-e8483-s001.pdf]

# Appendix

### Human ALPI deficiency causes inflammatory bowel disease and highlights a key mechanism of gut homeostasis

Marianna Parlato[1, 2], Fabienne Charbit-Henrion[1, 2, 3, 4], Jie Pan[5], Claudio Romano[2, 6], Rémi Duclaux-Loras[1, 2, 3], Marie-Helene Ledu[7], Neil Warner[5], Paola Francalanci[8], Julie Bruneau[3, 9], Marc Bras[10], Mohammed Zarhate[11], Bernadette Bègue[1, 2], Nicolas Guegan[1, 3], Sabine Rakotobe[1], 2, Nathalie Kapel[12], Paola De Angelis[8], Anne M Griffiths[5], Karoline Fiedler[5], Eileen Crowley[5], Frank Ruemmele[1, 2, 3, 4], Aleixo M. Muise[5, 13, 14,*], Nadine Cerf-Bensussan[1, 2, 3,*]

[1] INSERM, UMR1163, Laboratory of Intestinal Immunity and Institut Imagine, F-75015 Paris, France.
[2] GENIUS group from ESPGHAN.
[3] Université Paris Descartes-Sorbonne Paris Cité, F-75015 Paris, France.
[4] Assistance Publique-Hôpitaux de Paris, Hôpital Necker-Enfants Malades, Department of Pediatric Gastroenterology, Paris, France.
[5] SickKids Inflammatory Bowel Disease Center and Cell Biology Program, Research Institute, Hospital for Sick Children, Toronto, Ontario, Canada.
[6] Unit of Pediatrics, Department of Human Pathology in Adulthood and Childhood "G. Barresi", University of Messina, Viale Consolare Valeria, 98124, Messina, Italy.
[7] Department of Biochemistry, Biophysics and Structural Biology, Institute for Integrative Biology of the Cell (I2BC), CEA, UMR 9198 CNRS, Université Paris-Sud, Batiment 144, CEA Saclay, Gif-sur-Yvette, F-91191, France.
[8] Digestive Endoscopy and Surgery Unit and Pathology Unit Bambino Gesù Children Hospital, IRCCS, Piazza Sant'Onofrio 4, 00165 Rome, Italy.
[9] Department of Pathology, Necker-Enfants Malades Hospital, Assistance Publique-Hôpitaux de Paris, 75015 Paris, France.
[10] Bioinformatics platform, Université Paris-Descartes-Paris Sorbonne Centre and Institut Imagine, F-75015 Paris, France.
[11] Genomic Platform, INSERM UMR 1163, Paris Descartes Sorbonne Paris Cite University, Imagine Institute, 75015 Paris, France.
[12] Department of Functional Coprology, Pitié Salpêtrière Hospital, Assistance publique-Hôpitaux de Paris (AP-HP), Paris, France.
[13] Division of Gastroenterology, Hepatology, and Nutrition, Department of Pediatrics, University of Toronto, Hospital for Sick Children, Toronto, Ontario, Canada.
[14] Department of Biochemistry, Institute of Medical Science, University of Toronto, Toronto, Ontario, Canada.
* These authors share senior authorship

## Table of Content

**Appendix Fig S1:** BLAST sequence alignment and coordinates of ALPP........................................ 3
**Appendix Table S1:** Autosomal recessive and *de novo* variants identified by WES in Patient 1............... 4
**Appendix Table S2:** Autosomal Recessive and *de novo* variants identified by WES in Patient 2.............. 5

**Appendix Fig S1:** BLAST sequence alignment and coordinates of ALPP (entry code 1EW2)[1].

Sequence alignment Query= ALPP; Sbjct= ALPI

```
sp|P05187|PPB1_HUMAN Alkaline phosphatase, placental type OS=Homo sapiens
GN=ALPP PE=1 SV=2
Sequence ID: Query_182371Length: 535Number of Matches: 1
Related Information
Range 1: 23 to 516 Graphics Next Match Previous Match

Query  20   VIPAEEENPAFWNRQAAEALDAAKKLQPIQKVAKNLILFLGDGLGVPTVTATRILKGQKN  79
            +IP EEENP FWNR+AAEAL AAKKLQP Q  AKNLI+FLGDG+GV TVTA RILKGQK
Sbjct  23   IIPVEEENPDFWNREAAEALGAAKKLQPAQTAAKNLIIFLGDGMGVSTVTAARILKGQKK  82

Query  80   GKLGPETPLAMDRFPYLALSKTYNVDRQVPDSAATATAYLCGVKANFQTIGLSAAARFNQ  139
             KLGPE PLAMDRFPY+ALSKTYNVD+ VPDS ATATAYLCGVK NFQTIGLSAAARFNQ
Sbjct  83   DKLGPEIPLAMDRFPYVALSKTYNVDKHVPDSGATATAYLCGVKGNFQTIGLSAAARFNQ  142

Query  140  CNTTRGNEVISVMNRAKQAGKSVGVVTTTRVQHASPAGTYAHTVNRNWYSDADMPASARQ  199
            CNTTRGNEVISVMNRAK+AGKSVGVVTTTRVQHASPAGTYAHTVNRNWYSDAD+PASARQ
Sbjct  143  CNTTRGNEVISVMNRAKKAGKSVGVVTTTRVQHASPAGTYAHTVNRNWYSDADVPASARQ  202

Query  200  EGCQDIATQLISNMDIDVILGGGRKYMFPMGTPDPEYPADASQNGIRLDGKNLVQEWLAK  259
            EGCQDIATQLISNMDIDVILGGGRKYMF MGTPDPEYP D SQ G RLDGKNLVQEWLAK
Sbjct  203  EGCQDIATQLISNMDIDVILGGGRKYMFRMGTPDPEYPDDYSQGGTRLDGKNLVQEWLAK  262

Query  260  HQGAWYVWNRTELMQASLDQSVTHLMGLFEPGDTKYEIHRDPTLDPSLMEMTEAALRLLS  319
             QGA YVWNRTELMQASLD SVTHLMGLFEPGD KYEIHRD TLDPSLMEMTEAALRLLS
Sbjct  263  RQGARYVWNRTELMQASLDPSVTHLMGLFEPGDMKYEIHRDSTLDPSLMEMTEAALRLLS  322

Query  320  RNPRGFYLFVEGGRIDHGHHEGVAYQALTEAVMFDDAIERAGQLTSEEDTLTLVTADHSH  379
            RNPRGF+LFVEGGRIDHGHHE  AY+ALTE +MFDDAIERAGQLTSEEDTL+LVTADHSH
Sbjct  323  RNPRGFFLFVEGGRIDHGHHESRAYRALTETIMFDDAIERAGQLTSEEDTLSLVTADHSH  382

Query  380  VFSFGGYTLRGSSIFGLAPSKAQDSKAYTSILYGNGPGYVFNSGVRPDVNESESGSPDYQ  439
            VFSFGGY LRGSSIFGLAP KA+D KAYT +LYGNGPGYV   G RPDV ESESGSP+Y+
Sbjct  383  VFSFGGYPLRGSSIFGLAPGKARDRKAYTVLLYGNGPGYVLKDGARPDVTESESGSPEYR  442

Query  440  QQAAVPLSSETHGGEDVAVFARGPQAHLVHGVQEQSFVAHVMAFAACLEPYTACDLAPPA  499
            QQ+AVPL  ETH GEDVAVFARGPQAHLVHGVQEQ+F+AHVMAFAACLEPYTACDLAPPA
Sbjct  443  QQSAVPLDEETHAGEDVAVFARGPQAHLVHGVQEQTFIAHVMAFAACLEPYTACDLAPPA  502

Query  500  CTTDAAHPVAASLP  513
             TTDAAHP  + +P
Sbjct  503  GTTDAAHPGRSVVP  516
```

[1] Le Du MH, Stigbrand T, Taussig MJ, Menez A, Stura EA. Crystal structure of alkaline phosphatase from human placenta at 1.8 A resolution. Implication for a substrate specificity. J Biol Chem. 2001 Mar 23;276(12):9158-65.

**Appendix Table S1:** Autosomal recessive and *de novo* variants identified by WES in Patient1

| Gene | Variant | Protein | SIFT | MUTATION TASTER | POLYPHEN |
|---|---|---|---|---|---|
| ***de novo: SETD1A*** | 16 :30991333 C/A | p.Pro1409Gln | Tolerated | Polymorphism | Benign |
| ***de novo: ADAMTS14*** | rs758220536 | p.Arg451His | Deleterious | Disease causing | Probably damaging |
| **AR[1]: *ALPI*** | 2:233321394 G/A | p.Ala97Thr | Deleterious | Disease causing | Probably damaging |
| | 2:233322984 C/T | p.Ala350Val | Tolerated | Disease causing | Possibly damaging |
| **AR: *NWD1*** | 19:16918475 C/T | p.Thr1272Met | Tolerated | Polymorphism | Probably damaging |
| | rs191976059 | p.His843Gln | Tolerated | Polymorphism | Benign |
| **AR: *KRTAP5-4*** | rs200027081 | p.Val152Gly | not scored | not scored | not scored |
| | 11:1643184_TGAG CCACAGCCCCCA | frameshift | not scored | not scored | not scored |
| **AR: *TLN1*** | rs141770878 | p.Lys2115Glu | Deleterious | Disease causing | Probably damaging |
| | rs200664793 | p.Thr585Met | Tolerated | Disease causing | Possibly damaging |
| | rs144809355 | p.Ala2013Thr | Tolerated | Disease causing | Possibly damaging |
| **AR*: HGFAC*** | rs149401735 | p.Pro84Arg | Tolerated | Polymorphism | Benign |
| **AR: *TBX22*** | X:79277859 G/C | p.Glu31Gln | Tolerated | Polymorphism | Benign |
| **AR: *ARSF*** | rs764160465 | p.Thr299Met | Deleterious | Disease causing | Possibly damaging |
| **AR: *KIAA1875*** | rs540903279 | p.Glu1207Lys | not scored | not scored | Possibly damaging |

**Appendix Table S2:** Autosomal Recessive and *de novo* variants identified by WES in Patient2

| Gene | Variant | Protein | SIFT | MUTATION TASTER | POLYPHEN |
|---|---|---|---|---|---|
| ***de novo: ADRA1D*** | rs55915202 | p.Ala460Val | Tolerated | Disease causing | Benign |
| ***de novo: FKBP10*** | 17:39973373 | frameshift | not scored | not scored | not scored |
| ***de novo: FOXP2*** | 7:114271580 | 3 nt deletion in splice_acceptor | not scored | not scored | not scored |
| **AR: *ALPI*** | rs146257849 | p.Ala360Val | Tolerated | Disease causing | Probably damaging |
| | 2:233323584 C/T | p.Gln439X | not scored | not scored | not scored |
| **AR: *FLG*** | rs12750571 | p.Arg1437Cys | Tolerated | Polymorphism | Possibly damaging |
| | rs143382793 | p.Gly929Val | Tolerated | Polymorphism | Possibly damaging |
| **AR: *FRMPD1*** | rs200027081 | p.Val152Gly | Deleterious | Disease causing | Possibly damaging |
| | rs140778637 | p.Glu1519Ala | Deleterious | Disease causing | Possibly damaging |
| **AR: *MUC17*** | 7:100678206 C/T | p.Thr1170Met | Deleterious | Disease causing | Possibly damaging |
| | rs145956810 | p.Ser1788Leu | Deleterious | Disease causing | Possibly damaging |
| **AR: *NT5C3B*** | rs139142062 | p.Arg263His | Deleterious | Disease causing | Probably damaging |
| | 7:39992191 C/A | p.Ala11Ser | Tolerated | Disease causing | Benign |
| **AR: *PCDH9*** | rs149158184 | p.Ser1209Arg | Tolerated | Disease causing | Benign |
| | rs149470963 | p.Ser1017Arg | Tolerated | Disease causing | Benign |
| | rs139732295 | p.Val885Leu | Deleterious | Disease causing | Probably damaging |
| **AR: *FAT2*** | rs61743253 | p.Arg368Lys | Tolerated | Polymorphism | Possibly damaging |
| **AR: *RIOK1*** | rs55698032 | p.Arg114Gln | Tolerated | Disease causing | Benign |
| **AR: *STX8*** | rs55698032 | p.Arg141Gln | Tolerated | Disease causing | Benign |
