# Supplementary material for: Human ALPI deficiency causes inflammatory bowel disease and highlights a key mechanism of gut homeostasis
Source: EMBO Mol Med. 2018 Mar 22;10(4):e8483. doi: 10.15252/emmm.201708483 (PMC5887907; doi:10.15252/emmm.201708483)
Supplement: Supplementary file 2 — Expanded View Figures PDF [file EMMM-10-e8483-s002.pdf]

Expanded View Figures

P1

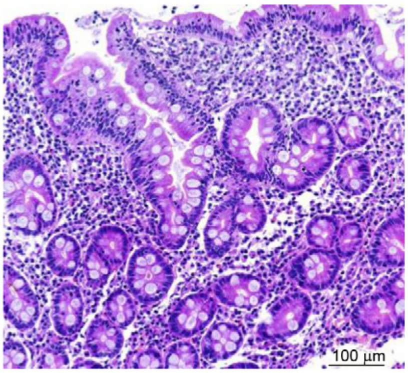

Figure EV1. Duodenal inflammation in ALPI-deficient patient P1.
Haematoxylin–eosin staining of duodenal biopsy from P1. Scale bar: 100 µm.

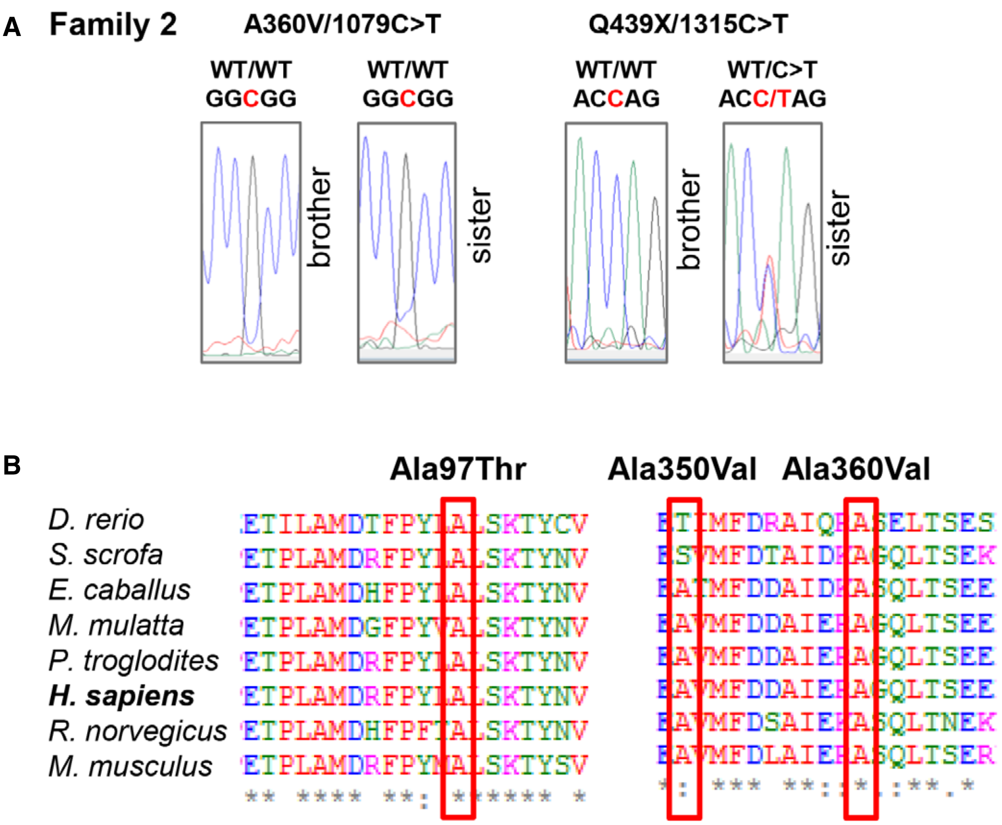

Figure EV2. Autosomal recessive ALPI deficiency.

A Confirmatory Sanger sequencing for siblings in Family 2.

B Multiple alignments of ALPI orthologs from different species using the Clustal Omega software. Residues altered by mutations in P1 and P2 are boxed in red. Conserved residues are indicated as follow: full identity (*), similar characteristics (:) (> 0.5 in the Gonnet PAM 250 matrix), weak similarities (.) (< 0.5 in the Gonnet PAM 250 matrix).

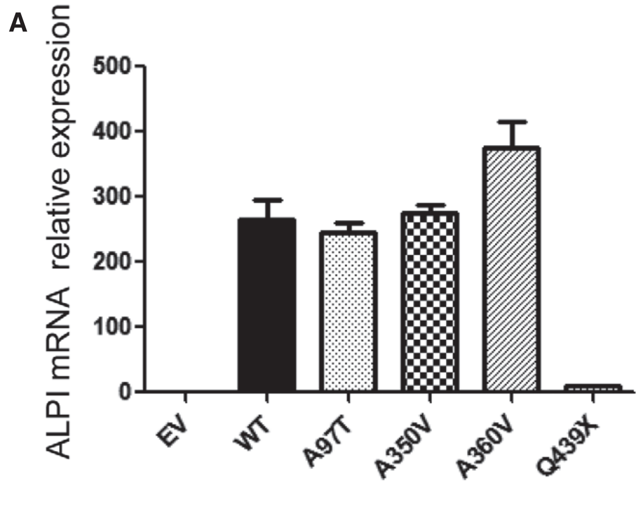

Figure EV3. mRNA ALPI expression in HEK293T cells.
WT ALPI and mutant expression by qRT-PCR.
Data are representative of three experiments and are expressed as mean ± SD.

© 2018 The Authors

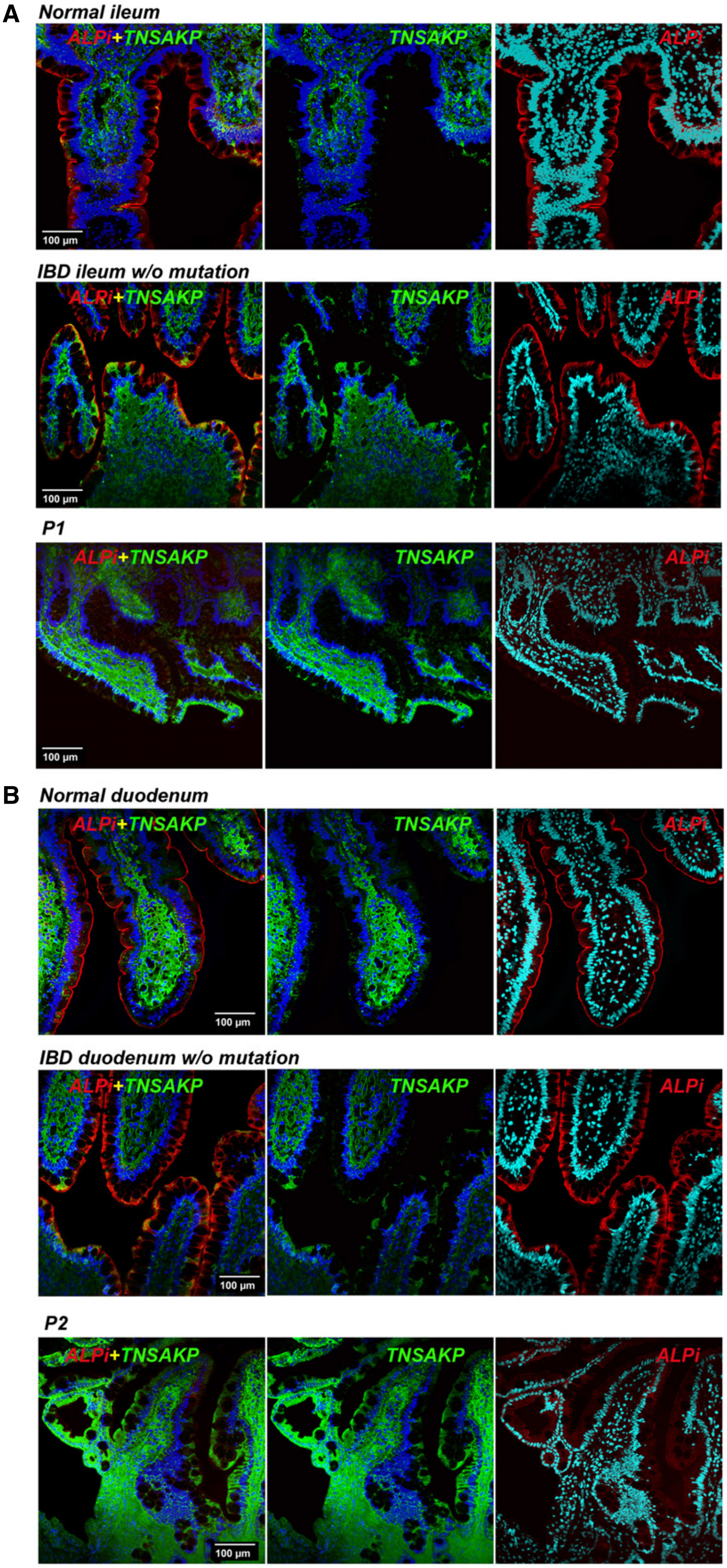

Figure EV4. TNAP expression in patients' small intestinal biopsies.

A, B Immunofluorescence microscopy of duodenum (A) and ileum (B) sections from P1 (A) and P2 (B) compared with normal or IBD controls. Sections were counterstained with blue RedDot2 dye for DNA and antibodies against ALPI alone (red), TNAP alone (green) or TNAP and ALPI (first column, merge staining). Magnification 20× (Scale bars: 100 µm).

© 2018 The Authors
