# Supplementary figures and images for: Human ALPI deficiency causes inflammatory bowel disease and highlights a key mechanism of gut homeostasis
Source: EMBO Mol Med. 2018 Mar 22;10(4):e8483. doi: 10.15252/emmm.201708483 (PMC5887907; doi:10.15252/emmm.201708483)

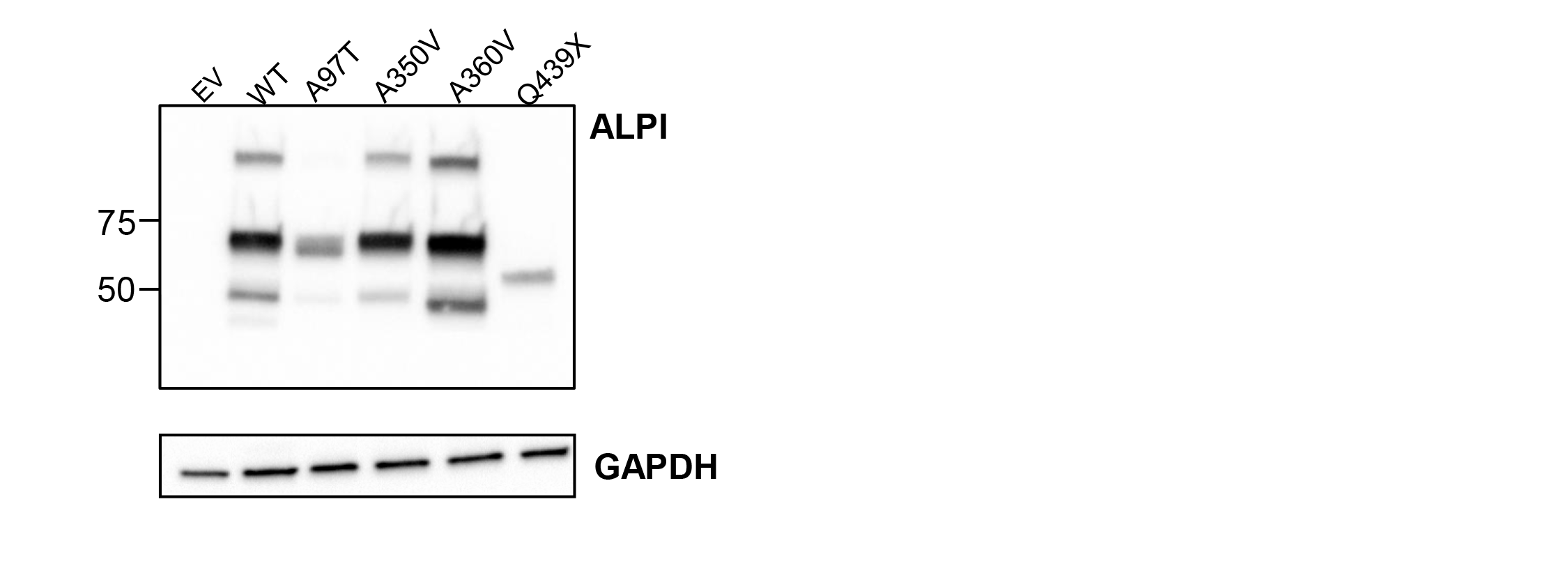

Supplement: Supplementary file 4 — Source Data for Figure 3 [file EMMM-10-e8483-s003.tif]
